# Dynamic Crosslinked Injectable Mussel-Inspired Hydrogels with Adhesive, Self-Healing, and Biodegradation Properties

**DOI:** 10.3390/polym15081876

**Published:** 2023-04-14

**Authors:** Ruixiao Wang, Liqun Liu, Xiang He, Zongmei Xia, Zhenjie Zhao, Zhenhao Xi, Juan Yu, Jie Wang

**Affiliations:** 1 State Key Laboratory of Chemical Engineering, East China University of Science and Technology, Shanghai 200237, China; y30200220@mail.ecust.edu.cn (R.W.); leoliu0811@163.com (L.L.); y30190862@mail.ecust.edu.cn (X.H.); xiazongmei1999@163.com (Z.X.); zhaozhenjieemail@163.com (Z.Z.);; 2 Shanghai Key Laboratory of Multiphase Materials Chemical Engineering, East China University of Science and Technology, Shanghai 200237, China

**Keywords:** tissue adhesive, in situ hydrogel, hyaluronic acid, carboxymethyl chitosan

## Abstract

The non-invasive tissue adhesives with strong tissue adhesion and good biocompatibility are ideal for replacing traditional wound treatment methods such as sutures and needles. The self-healing hydrogels based on dynamic reversible crosslinking can recover their structure and function after damage, which is suitable for the application scenario of tissue adhesives. Herein, inspired by mussel adhesive proteins, we propose a facile strategy to achieve an injectable hydrogel (DACS hydrogel) by grafting dopamine (DOPA) onto hyaluronic acid (HA) and mixing it with carboxymethyl chitosan (CMCS) solution. The gelation time and rheological and swelling properties of the hydrogel can be controlled conveniently by adjusting the substitution degree of the catechol group and the concentration of raw materials. More importantly, the hydrogel exhibited rapid and highly efficient self-healing ability and excellent biodegradation and biocompatibility in vitro. Meanwhile, the hydrogel exhibited ~4-fold enhanced wet tissue adhesion strength (21.41 kPa) over the commercial fibrin glue. This kind of HA-based mussel biomimetic self-healing hydrogel is expected to be used as a multifunctional tissue adhesive material.

## 1. Introduction

Skin is the largest organ of the human body and the first protective barrier against external harm [1,2]. Bacterial infection of wounds can significantly compromise the healing process [3,4]. Traditional treatments are painful and have the potential risk of wound infection and secondary injury [5]. Nowadays, medical tissue adhesives have the advantages of convenient operation, no need for secondary surgery, and adaptability to irregular wounds [6]. Hydrogels are kinds of polymers with 3D networks that provide cells with a similar environment mimicking the extracellular matrix of native tissues [7,8], absorbing excess tissue exudates, protecting wounds from infection, and removing free radicals. Therefore, they are ideal materials for medical adhesives [9,10]. Injectable hydrogels are fabricated and classified as physical or chemical hydrogels based on the mechanism of gelation and formulated structure upon crosslinking [11,12]. Moreover, the injectable hydrogel can be a competitive candidate for wound dressing due to its rapid and reversible sol–gel transition behavior, which helps to fill the irregular wound area completely by minimally invasive injection [13].

Many hydrogel-forming biomaterials have been proposed, including collagen, chitosan, hyaluronate, fibrin, and various block copolymers. Hyaluronic acid (HA) widely exists in the human body and is the main component of the extracellular matrix [14]. Its biocompatibility and biodegradability have been widely recognized [15]. In addition, HA has a certain regulatory effect on the diffusion and transport of protein, vascular permeability, and water electrolyte [14,16]. HA helps fibroblasts and endothelial cells reorganize, proliferate, and migrate [17]. Furthermore, it promotes angiogenesis during wound healing [18]. HA can form a viscoelastic solution in the dermis to fill the gap between collagen fibers so it is often used as a moisturizing ingredient in skin care products and wound adhesive [19]. The hydrogel prepared from HA can absorb wound exudate and blood, temporarily acting as a physical barrier to the skin [20,21]. For these reasons, HA is considered to be a very attractive raw material for tissue adhesives [22]. In addition to HA, other natural substances are also commonly used to produce biocompatible hydrogels. Carboxymethyl chitosan (CMCS) is an important, water-solution derivative of chitosan, which contains cationic and anionic groups. CMCS is widely used in drug delivery and wound treatment because of its good biocompatibility, nontoxicity, and biodegradability [7]. CMCS-based hydrogels possess good abilities of antibacterial and prevent wound infection during wound healing [23].

However, some hydrogels’ weak adhesion to wet surfaces limits their clinical application [6,24]. Inspired by the mussel’s outstanding adhesive ability to various substrates in a moisture environment, many mussel adhesive protein mimetic hydrogels are designed [25]. The mussel adhesive proteins contain a large amount of 3,4-dihydroxy phenyl-L-alanine, which gives them high adhesion energy [26,27,28]. This is mainly because the catechol group can trigger hydrogen bonds, covalent bonds, π–π and cation–π interactions [29]. Besides, the hydroxyl group on catechol can form bidentate chelating interaction with the substrate, which greatly improves the interaction force of hydrogen bonds so that it has strong adhesion to a variety of surfaces [28]. Therefore, the structure with catechol was often introduced into the hydrogel to improve its adhesion and mechanical strength, and a series of mussel bionic adhesives were studied and developed [30,31].

Here, a dynamic crosslinked injectable hydrogel (DACS hydrogel) was prepared by mixing dopamine-grafted oxidized hyaluronic acid (DAHA) and CMCS solution based on Schiff base reactions. The effects of catechol group substitution degree, DAHA concentration, and CMCS concentration on the gelation time and rheological properties of hydrogels were studied. To explore the adhesion properties of DACS hydrogels, the lap shear tensile test of porcine skin was carried out. The self-healing properties of hydrogels were tested by macroscopic observation and rheology, and the degradation ability in vitro of DACS hydrogels was tested. Meanwhile, the live-dead assay against L929 cells in vitro of DACS hydrogels was investigated.

## 2. Materials and Methods

### 2.1. Materials

Hyaluronic acid (HA, Mw = 170 kDa), dopamine hydrochloride (DA, 99%), and carboxymethyl chitosan (CMCS, 99%) were obtained from Shanghai Macklin Biochemical Co., Ltd. (Shanghai, China) Sodium periodate and glycol were supplied by Shanghai Aladdin Bio-Chem Technology Co., Ltd. (Shanghai, China) All the chemicals were of analytical grade and were used without further purification.

### 2.2. Synthesis of Aldehyde-Modified Sodium Hyaluronate (AHA)

Firstly, 1.00 g of HA was dissolved in 100 mL of ultrapure water, and 0.56 g sodium periodate was added and stirred for 24 h under dark conditions at room temperature. After the reaction, 1 mL of ethylene glycol was added to the solution and stirred for an additional hour to neutralize the excess sodium periodate. Finally, the solution was purified by dialysis (MWCO: 8000 Da) using a large amount of ultrapure water for 2 days (3 times a day, 1 L each time). The final product was lyophilized and kept in a dryer for further use. The chemical structure of AHA was characterized by ^1^H NMR (Ascend 600 M, BRUKER, Ettlingen, Germany), and FTIR (Nicolet 6700, Thermo Nicolet Corporation, Madison, WI, USA).

### 2.3. Synthesis of Dopamine-Conjugated AHA (DAHA)

The fabrication of DAHA was improved according to the preparation method in the literature [32]. A total of 1.00 g of AHA was dissolved in 100 mL of phosphate-buffered saline (PBS, pH = 5.0) followed by adding DA of equal molar (AHA/DA = 1:1) and stirring for 10 h with the protection of nitrogen and under dark conditions at room temperature. The specific preparation scheme is shown in Table S3. The reaction solution was dialyzed against ultrapure water for 3 days. During the dialysis, the dialysate was measured by UV-vis (SHIMADZU UV-1900i, Shimadzu, Kyoto, Japan) to ensure that the free DA was removed completely. The final product was lyophilized and kept in a dryer for further use. The chemical structure of DAHA was characterized by ^1^H NMR (Ascend 600 M, BRUKER, USA), FTIR (Nicolet 6700, Thermo Nicolet Corporation, Madison, WI, USA), and UV-vis (SHIMADZU UV-1900i, Shimadzu, Kyoto, Japan). The absorbance of DAHA solution with a known concentration at 280 nm was measured, and the standard curve of DA was calculated using UV-vis.

### 2.4. Formation of DACS Hydrogels

The preparation of DACS hydrogels mainly included two steps. Firstly, DAHA and CMCS were dissolved in PBS (pH = 5.0), respectively. Secondly, 1 mL DAHA solution and 1 mL CMCS solution were taken into the same bottle, gently mixed, and gelled quickly, which was named DACS hydrogel. The final concentration of DAHA and CMCS was 3 wt% and 2.5 wt%, respectively.

### 2.5. Characterization of Hydrogels

The time required for the two gel precursor solutions to gradually change from the free flow state at the beginning of contact to the time when the solutions do not flow anymore is the gelation time of DACS hydrogels. The gelation time of the hydrogels was calculated according to the vial inverted method, which was monitored by inversing test bottles every 5 s. DACS hydrogels were placed in a 37 °C incubator for 12 h and lyophilized (self-weight, *W*0), then immersed in PBS (pH = 7.4). At regular intervals, swollen hydrogels were weighed after gently removing the surface-adhering solutions using filter paper and recorded as *Wt*. The swelling ratio was calculated by the formula $r=\left(Wt-W0\right)/W0$. When the mass of the wet gel remained unchanged, the swelling equilibrium of the hydrogel was reached. Besides, we analyzed the concentration of DAHA and CMCS at the gelation time and swelling properties of the hydrogel. All experiments were performed in triplicate.

### 2.6. Rheological Properties

Rheological tests of the hydrogels were performed using a 20 mm plate and a 1.0 mm gap in a rheometer (HAAKE Mars 60, Karlsruhe, Baden-Württemberg, Germany). The hydrogels were formed directly on the plate at 37 °C. Before the time sweep tests, the linear viscoelastic region was determined by the strain amplitude sweep in the range of 0.1–1000% at 1 Hz. The time sweep test was used to get the crosslinking state inside the hydrogel (the strain was set to 1%, and the frequency was set to 1 Hz). The degree of substitution of DAHA in the hydrogel system was changed, and its effect on the rheological properties of the hydrogel was analyzed.

### 2.7. Self-Healing Test

The hydrogels were stained with methylene orange (orange dyes) and rhodamine B (red dyes), respectively. Then, the hydrogels were cut into two pieces, and the cut interfaces were contacted without external intervention for healing. HAAKE Mars 60 (Karlsruhe, Baden-Württemberg, Germany) rheometer was used to test the self-healing ability of the hydrogel to external action with alternating applied strain. The test was carried out at 1 Hz frequency. Specifically, after a weak 1% stress of amplitude oscillatory stress for 240 s, the stress would increase to 700% suddenly and remain for 120 s.

### 2.8. Tissue Adhesive Strength Test

For qualitative testing, the DAHA solution (300 μL) was applied onto the bottom of 50 mL beaker, and CMCS solution (300 μL) was coated onto the surface of plastic, metal, and ceramic materials. Then, the beaker was quickly stuck to the designated area of the substrate. To adhere, the specimen was kept under 15 g weights for 30 min at room temperature.

The adhesive strength of DACS hydrogel to other substrate materials, such as plastic and glass, was tested. The method was investigated by lap shear test according to the ASTM F2255-05 standard. The surface of the aluminum plate was polished, and the plates with different substrate materials were glued firmly with cyanoacrylate glue on aluminum plates. The DAHA solution (200 μL) was added to 1 piece, and CMCS solution (200 μL) was applied to the surface of another piece. Then, the 2 plates were stacked up quickly and pressed tightly with clips at 37 °C for 2 h (bonding area is 10 × 25 mm^2^). The adhesive strength was assessed using a universal testing machine (HengYi, HY-0580 Shanghai Hengyi Precision Instrument Co., Ltd., Shanghai, China) at room temperature with a 100 N load cell at a strain speed of 5 mm/min. Adhesion strength was calculated on a formula of dividing the maximum load force by bonding area.

The tissue adhesion strength of DACS hydrogel to porcine skin was also tested. The wet porcine skin was glued with cyanoacrylate glue on aluminum plate, and the test method of adhesion strength was the same as above. All measurements were repeated three times.

### 2.9. In Vitro Degradation Test

Lyophilized gels (weighted as *W*0) were completely immersed in PBS (pH = 7.4). After the hydrogels reached full swelling (about 12 h later), hyaluronidase (0.05 mg/mL) was added to the buffer solution. At the set time point, the gel was taken out, lyophilized, and weighed as *Wt*. The DACS hydrogel degradation rate was according to the following formula: $\frac{Wt-W0}{W0}*100\%$.

### 2.10. In Vitro Cytotoxicity Test

An extraction method was utilized to evaluate the cytotoxicity of DACS hydrogel using L929 cells. DACS hydrogel (1 mL 3.0 wt% DAHA, 1 mL 2.5 wt% CMCS) was sterilized by UV irradiation and then immersed in a cell culture medium for 72 h. After that, the solution was filtered to remove bacteria. Subsequently, the L929 cells were seeded on the cover glass inside the 12-well culture plate at a density of 5.0 × 10^4^ cells/well. After cell attachment, the cell culture medium was replaced with the extracted hydrogel media at 37 °C in an incubator with 5% CO_2_ for 3 days. On the 1st, 2nd, and 3rd day of the culture process, the cell viability was measured using an MTT assay. Cells were cultured in a medium with the tissue culture polystyrene dish (TCPS) as control. The tests were carried out in triplicate.

## 3. Results

### 3.1. Synthesis and Characterization of DAHA

The AHA was obtained through the oxidation reaction of the hydroxyl (-OH) on the HA backbone (Figure S1). Its oxidation degree (0.55–38.32%) and molecular weight (Mw, 1724580–18961) were shown in Tables S1 and S2, respectively. DAHA was obtained by grafting DA onto AHA through Schiff base reactions (Figure S2). The effects of the oxidation degree of AHA, raw material ratio (DA/AHA), and reaction time on the substitution degree (DS) of DA were investigated. The degree of DA substitution in DAHA products obtained under different preparation conditions is shown in Table S3. The highest DS of DA obtained in the experiment was 88.5%, which was much higher than that in similar studies [33]. The products were characterized by FTIR (Figure S3), ^1^H NMR (Figure S4) and UV-vis (Figure S5). The results confirmed that dopamine was successfully grafted onto the AHA molecular chains. In the reaction process, DAHA with different catechol content could be synthesized by adjusting the feed ratio of DA and AHA to prepare hydrogel with different properties.

### 3.2. Preparation of DACS Hydrogel

**Figure 1:**
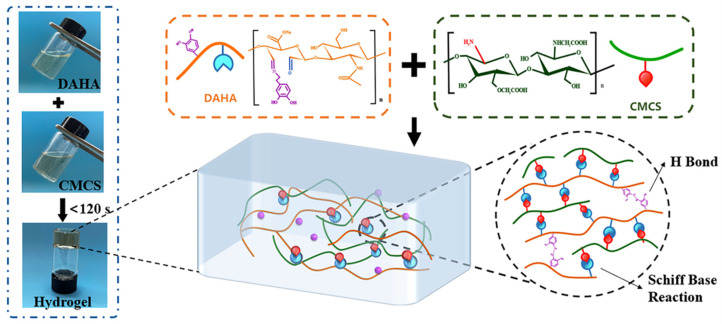
Schematic diagram of crosslinking within DACS hydrogels by mixing DAHA and CMCS solutions.

The DACS hydrogel was prepared by only a simple method of mixing the DAHA solution and CMCS solution by magnetic stirring (Figure 1). Compared with the chemical crosslinking process using glutaraldehyde and other crosslinking agents, the preparation of hydrogels by dynamic bond crosslinking is a mild, safe, and efficient gelling method. As shown in Figure 1, the crosslinking of the hydrogel is based on Schiff base reactions and hydrogen bonds. The amino group (-NH_2_) in CMCS reacts with the aldehyde group (-CHO) in DAHA to produce the imine bond (-CH=N-), which is the first covalent crosslinking network. In addition, hydrogen bonding between phenolic hydroxyl groups on DA grafted on DAHA, and hydrogen bonding within and between HA and CMCS molecules are also important forces in crosslinking networks [34].

### 3.3. Mechanical Properties of DACS Hydrogel

**Figure 2:**
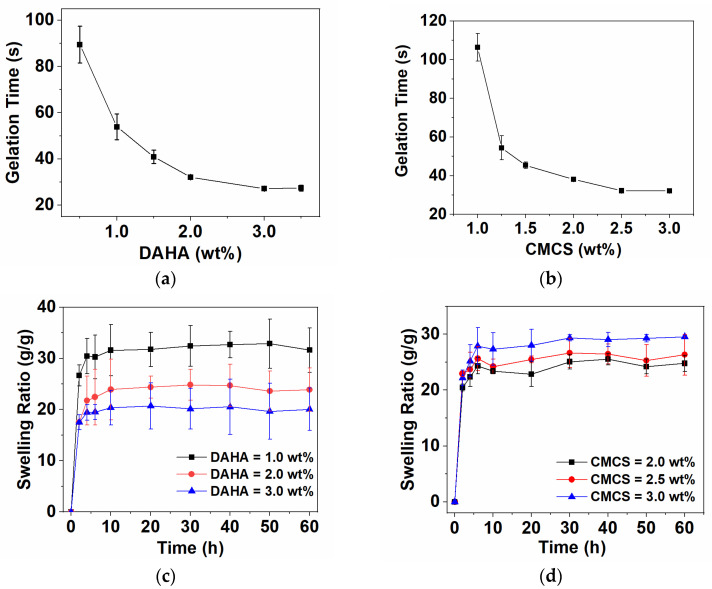
Effect of gelation time by DAHA and CMCS concentrations. [DAHA] = 0.5–3.5 wt%, [CMCS] = 2.5 wt% (**a**), [CMCS] = 1.0–3.0 wt%), [DAHA] = 3 wt% (**b**); Effect of swelling ratio by DAHA and CMCS concentrations. [DAHA] = 1.0–3.0 wt%, [CMCS] = 2.5 wt% (**c**), [CMCS] = 2.0–3.0 wt%, and [DAHA] = 3 wt% (**d**).

To reach the application of tissue adhesive in emergency surgery, wound repair, and other fields, hydrogels need to achieve rapid and controllable in situ gelation. The gel-forming time of hydrogel was studied by the vial pouring method. The gelation time of the DACS hydrogels could be easily changed with different concentrations of DAHA and CMCS. As shown in Figure 2a,b, when the CMCS concentration was kept at 2.5 wt%, the gelation time decreased from 90 s to 27 s with the increase of DAHA concentration from 0.5 wt% to 3.5 wt%; when the DAHA concentration was kept at 3.0 wt%, the gelation time decreased from 102 s to 32 s with the increase of CMCS concentration from 1.0 wt% to 3.0 wt %. That is because the increase of -CHO or -NH_2_ in the system leads to the increase of crosslinking sites and the acceleration of gelation speed. Therefore, when the concentration increases, the determination of gelation time becomes more accurate, and the error becomes smaller. However, when the concentration of DAHA and CMCS was increased to a certain value, the gelation time changed little. Considering that too fast gelling speed is not conducive to the full mixing of raw materials and the application of the gel, the concentration of DAHA and CMCS was chosen as 3.0 wt% and 2.5 wt%, respectively, in the subsequent experiments.

Since hydrogel is used on patients’ skin wounds, it is hoped that wound dressings can absorb wound exudates. Swelling behavior is one of the basic characteristics of hydrogels. The properties of the hydrogels with different concentrations of DAHA and CMCS were also investigated by the swelling ratio test. As shown in Figure 2c,d, when the concentration of DAHA or CMCS was changed, the swelling degree of DACS hydrogel increased rapidly, and the hydrogels reached the swelling equilibrium in about 10 h. Even for 60 h, the hydrogel had no obvious mass loss. When the concentration of DAHA increased from 1.0 wt% to 3.0 wt%, the swelling degree gradually decreased from 30- to 20-fold in self-weight. This may mainly be attributed to the increased sites of Schiff base reactions with CMCS when the concentration of DAHA increased, making the crosslinking density of the hydrogel higher. However, with the concentration of CMCS increasing from 2.0 wt% to 3.0 wt%, the equilibrium swelling degree of hydrogel increased from 23- to 28-fold in self-weight. The main reason is that more -NH_2_ and -COO- had electrostatic interaction in the reaction system. Additionally, the crosslinking degree of the hydrogel system dominated by electrostatic interaction with relatively weak bond energy was also relatively weak. In addition, with the increase in CMCS concentration, the long-chain macromolecules in the system increased, and the steric hindrance increased accordingly, which made the crosslinking density of the hydrogel lower.

### 3.4. Rheological Properties of Hydrogels

**Figure 3:**
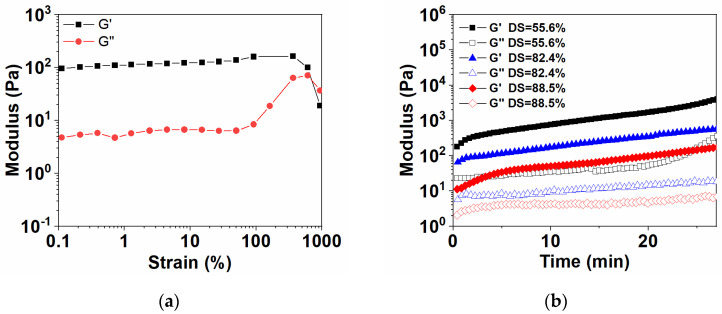
Rheometric tests of DACS hydrogels. (**a**) The amplitude sweep tests and (**b**) the time sweep tests at a frequency of 1 Hz and a strain of 1%.

The linear viscoelastic region (LVR) of hydrogels was determined by the oscillation mode of the rheometer, as shown in Figure 3a. The LVR of DACS hydrogels is 0.1–600%, and the maximum strain that hydrogels could bear without system damage was 600%, which was much higher than other hydrogels of the same type [29].

DACS hydrogels were prepared by mixing the solutions of CMCS and DAHA with different degrees of substitution, and the time sweep rheological experiment was conducted at a constant strain (1%) to ensure that the test conditions stayed within the linear viscoelastic region, as shown in Figure 3b. With the further increase of DAHA substitution degree (from 55.6% to 88.5%), the storage modulus (G’) of DACS hydrogels decreased in turn, indicating that the mechanical properties and hardness of hydrogel decreased. This was because the gelation of DACS hydrogels mainly depended on the Schiff base reactions between DAHA and CMCS. Meanwhile, the preparation of DAHA was also achieved by Schiff base reactions between DA and AHA. Therefore, the preparation of DAHA was competitive with the gelation of DACS hydrogels. It was inferred that when the degree of dopamine substitution increased, the steric hindrance of DAHA also increased. So, the crosslinking effect of hydrogel was correspondingly weakened, and its mechanical strength was also reduced.

### 3.5. Macroscopic Injectable and Self-Healing Experiments

**Figure 4:**
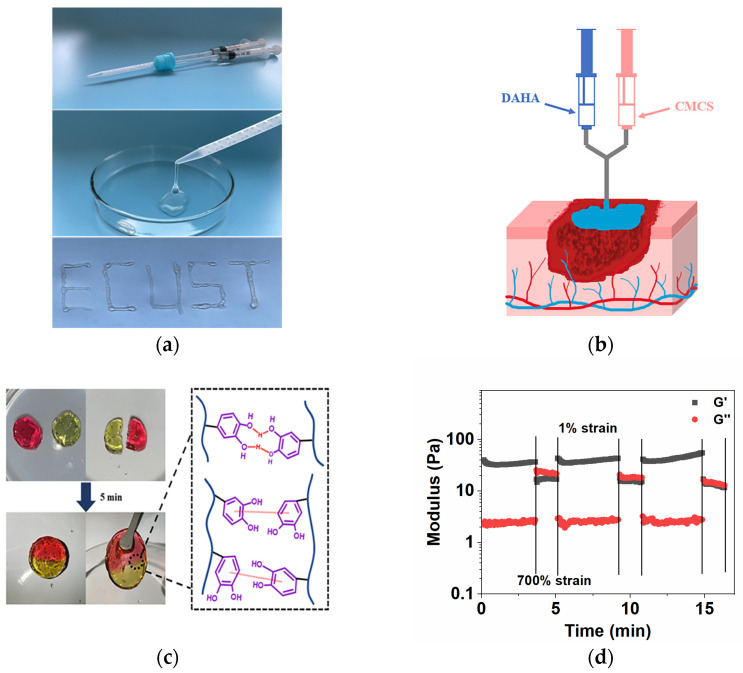
(**a**) Evaluation of injectability of the DACS with a double barrel spiral needle; (**b**) Schematic diagram of DACS hydrogel used for the wound; (**c**) Self-healing ability of DACS hydrogels and schematic diagram of the self-healing process; and (**d**) Rheological properties of DACS hydrogels under variable external strain (1–700%).

DACS hydrogel has good injectability. It can be injected with a double-barrel spiral syringe (Figure 4a). Moreover, the prefabricated hydrogel can still be injected with a 16 G (38 mm) needle through a syringe (Figure 4a). Because of its injectable properties, the hydrogel can be applied to irregular wound shapes and has a great application prospect in emergencies (Figure 4b). At present, most injectable hydrogels need to strictly control the gelation time to prevent the crosslinked hydrogels from blocking the needle. DACS hydrogel could be injected even after gelation. This is because the shear thinning effect will occur under the action of external force, and the gel state will transfer into a fluid state spontaneously. More importantly, DACS hydrogels can return to the gel state after the external force disappears, which is injectable self-healing properties. This characteristic is of great significance for the application of wound repair.

Self-healing hydrogels could inject into irregular wounds and recover to form a complete hydrogel, which is necessary for wound healing [35]. To visually measure the self-healing capability of the initial hydrogel, the macroscopic self-healing performance is shown in Figure 4c. After treatment, it was observed that the 2 halves of the hydrogel without any intervention could form a whole hydrogel in 5 min. The bonding part could achieve perfect fusion without any cracks, and the interface had a color transition. The results indicated that hydrogels have excellent self-healing properties. The mechanism of DACS hydrogel’s self-healing is shown in Figure 4c.

As shown in Figure 4d, the DACS hydrogels were tested by strain alternated rheological between 1% and 700% to further investigate its self-healing performance. At 1% strain, the structure of hydrogel was stable, and the storage modulus G’ was significantly higher than the loss modulus (G’’). When the hydrogel was at the high strain value of 700%, the hydrogel showed shear thinning characteristics, G’’ was higher than G’, and the hydrogel network structure was destroyed. When the applied strain was released, G’ immediately returned to the initial state and reappeared as a solid state (G’ > G’’). G’ and G’’ changed synchronously with the alternating change of strain, and this process could be repeated many times. The results of strain alternated sweep further showed that DACS hydrogels had an excellent self-healing performance.

### 3.6. Adhesive Strength Test

**Figure 5:**
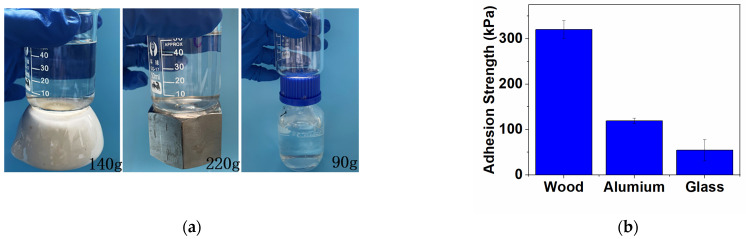
(**a**) Surface adaptability of DACS hydrogels: ceramic, metal, and plastic; (**b**) Average adhesion strength on wood, metal, and glass.

In this study, the adaptability of DACS hydrogels to different material surfaces was preliminarily investigated through the adhesion between the hydrogels and different surfaces. As shown in Figure 5a, only 600 μL hydrogels were needed to firmly bond 140 g of ceramic, 220 g of metal, and 90 g of plastic. This is mainly because the catechol group in the hydrogel can form strong physical and chemical interactions with the surface of each substrate [36].

The test method was used to measure the adhesive strength of DACS hydrogels on wood, aluminum plate, and glass surface. It could be seen from Figure 5b that the adhesive strength of the hydrogels on the wood surface could reach 320 kPa, which was probably due to the hydrogen bonds between a large number of amino groups on the wood surface and catechol groups in the hydrogels. In addition, the adhesion strength of the hydrogels in aluminum plate and glass could also reach 119.31 kPa and 54.56 kPa, respectively. The excellent surface adaptability proved that the DACS hydrogels could be used for the adhesion of a variety of surfaces.

### 3.7. In Vitro Cytotoxicity of DACS Hydrogels

**Figure 6:**
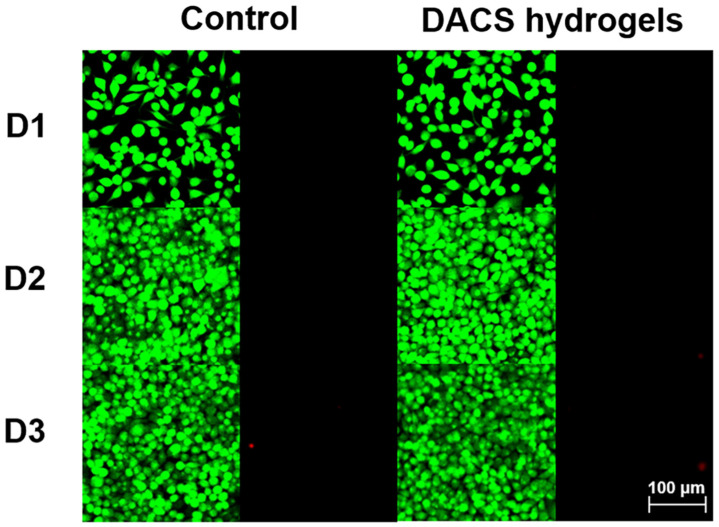
The fluorescence image of L929 mouse fibroblast cells cultured with control and DACS hydrogels leaching solution on the first (D1), second (D2), and third day (D3), respectively.

Since DACS hydrogel has good adhesive properties and injectability, we hope it can be used as a tissue adhesive, so it should be non-toxic. To confirm this, we used murine fibroblast cells (L929) as model cells and tested the biocompatibility of our hydrogels by Live/Dead staining. The hydrogels were incubated in a cell culture medium for 72 h at 37 °C, which were used for cell culture (L929 cells) after leaching. The Live/Dead staining was used to visually observe the cell biocompatibility, in which living cells with intact membranes were stained with calcein-AM (green channel), and dead cells was labeled with PI (red channel). It was found that the majority of L929 cells presented normal morphology, and the cells treated with the hydrogels exhibited a similar proliferation trend as the control group (Figure 6). These results strongly indicated that DACS hydrogels are non-toxic and can be further used in tissue adhesion.

### 3.8. Tissue Adhesive Strength Test

**Figure 7:**
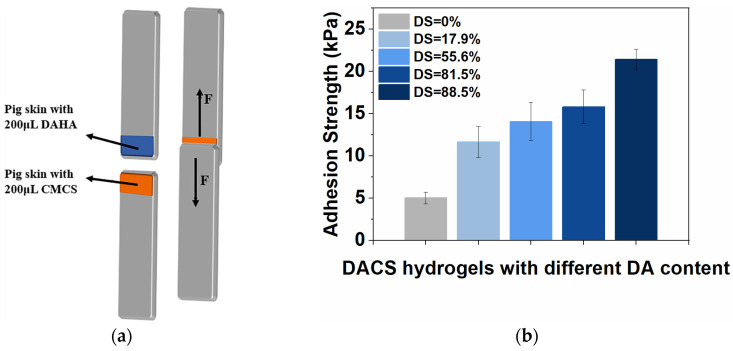
(**a**) The schematic diagram of lap-shear test of DACS hydrogels on porcine skin; (**b**) The average adhesion strength of DACS hydrogels with different DA content (17.9%, 55.6%, 81.5%, and 88.5%) on porcine skin tissue surfaces.

The tissue adhesion property of the DACS hydrogels was evaluated by the adhesion strength of the hydrogel on the surface of porcine skin (Figure 7a). Hydrogels with different DA substitution degrees were prepared, and their effects on the adhesive strength of hydrogel were also explored. The results were shown in Figure 7b. It could be seen that when the DA substitution degree increased from 0% to 17.9%, the adhesive strength of the hydrogel increased from 5.03 kPa to 11.4 kPa. With the degree of substitution gradually increased to 88.5%, the average adhesion strength of the hydrogel increased from 11.4 kPa to 21.41 kPa. Compared with the adhesion performance of commercial fibrin glue (5 kPa), the adhesion strength of DACS hydrogels could reach 2–4 times [9,37].

### 3.9. In Vitro Degradation Test

**Figure 8:**
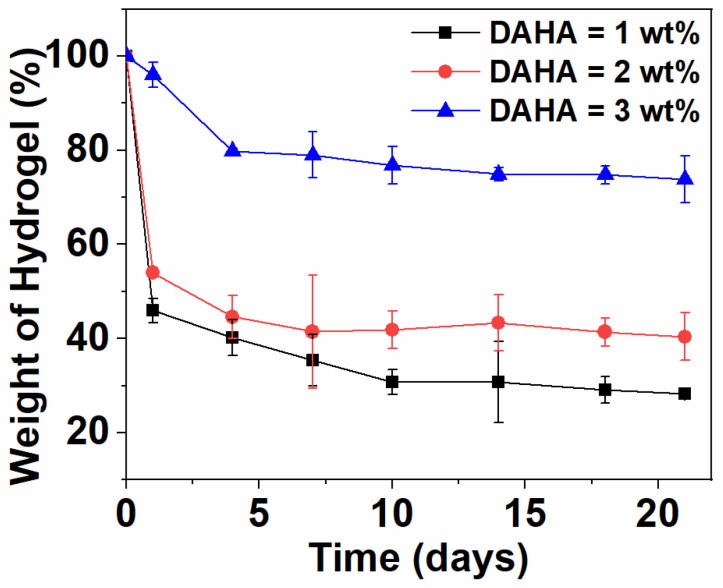
In vitro degradation of DACS hydrogels with different concentrations of DAHA (1.0–3.0 wt%).

The degradation behavior of a hydrogel is the key to determining the frequency of dressing replacement in the process of wound healing. Frequent replacement and hard removal of dressing may increase the cost and lead to secondary wound injury. The degradation rate of biomaterials relies on the ingredient, physicochemical properties, and biological conditions. Figure 8 shows the degradation curve of DACS hydrogels prepared with different concentrations of DAHA in a hyaluronidase solution. It could be found that all the hydrogels showed significant weight loss. When the hydrogel was degraded for 7 days, the maximum mass loss could reach 40%, and the maximum initial weight loss within 21 days was 74%. With the increase in DAHA concentration, the degradation rate of hydrogel slowed down. The weight loss rate of the hydrogel decreased significantly after the degradation time exceeded 10 days. By changing the concentration of DAHA, the degradation rate of the hydrogel can be adjusted and adapted to different wound healing cycles. Therefore, DACS hydrogels have good biodegradation performance and can be regulated, which meets the requirements of injectable self-healing tissue adhesive.

## 4. Conclusions

In this study, we design the DACS hydrogels in situ for accelerating wound healing through dynamic crosslinking of Schiff base and hydrogen bonds between DAHA and CMCS. The swelling degree of the hydrogel was stable without mass loss within 60 h. By controlling the substitution degree of dopamine and the concentration of raw materials, the gelation time of the hydrogel could be controlled (from 102 s to 27 s). Moreover, the controllable adjustment and balance of injectable and self-healing properties and high mechanical strength and bonding strength of the hydrogel can be reached. The hydrogels had controllable degradation rates, so they can adapt to different wound-healing cycles. The hydrogel exhibited strong adhesion to various substrates, such as wood, aluminum, glass, and porcine skin, and it also exhibits good cytocompatibility in vitro. These features revealed that hydrogels have a good application prospect in biomedicine.

## Data Availability

No applicable.

## Supplementary Materials

The following supporting information can be downloaded at: https://www.mdpi.com/article/10.3390/polym15081876/s1, Figure S1: Synthesis route of AHA; Table S1: Reaction conditions for oxidation of native hyaluronic acid with the corresponding degrees of oxidation. Table S2: GPC results of HA and AHA. Table S3: Synthesis of DAHA with different degrees of substitution (DS); Figure S2: Synthesis route of DAHA; Figure S3: Infrared spectra of HA, AHA, and DAHA; Figure S4. ^1^H NMR spectra of DA, HA, AHA, and DAHA; Figure S5: (a) UV-vis spectra of DAHA, (b) The absorbance of a series of dopamine hydrochloride solutions with different concentrations (ranging from 50–200 μg/mL) at 280 nm wavelength. (c) The standard curve was generated by measuring the absorbance of a series of dopamine hydrochloride solutions.
