# Supplementary material for: Dynamic Crosslinked Injectable Mussel-Inspired Hydrogels with Adhesive, Self-Healing, and Biodegradation Properties
Source: Polymers (Basel). 2023 Apr 14;15(8):1876. doi: 10.3390/polym15081876 (PMC10143368; doi:10.3390/polym15081876)
Supplement: Supplementary file 1 [file polymers-15-01876-s001.zip › Highlights.pdf]

Highlights

• The dynamic crosslinked hydrogel reflects injectable and self-healing properties.

• The mussel-inspired hydrogel possesses enhanced wet tissue adhesion.

• DACS hydrogel has good biocompatible and biodegradation properties.
