# Supplementary material for: Dynamic Crosslinked Injectable Mussel-Inspired Hydrogels with Adhesive, Self-Healing, and Biodegradation Properties
Source: Polymers (Basel). 2023 Apr 14;15(8):1876. doi: 10.3390/polym15081876 (PMC10143368; doi:10.3390/polym15081876)
Supplement: Supplementary file 1 [file polymers-15-01876-s001.zip › SI 0404.pdf]

Supporting Information

Dynamic crosslinked injectable mussel-inspired hydrogels with adhesive, self-healing and biodegradation properties

Ruixiao Wang[1], Liqun Liu[1], Xiang He[1,2], Zongmei Xia[1], Zhenjie Zhao[1], Zhenhao Xi [1,2], Juan Yu[1], and Jie Wang[1*]*

[1]State-Key Laboratory of Chemical Engineering, East China University of Science and Technology, Shanghai 200237, China

[2]Shanghai Key Laboratory of Multiphase Materials Chemical Engineering, East China University of Science and Technology, Shanghai 200237, China.

*Email: zhhxi@ecust.edu.cn (Zhenhao Xi); jiewang2010@ecust.edu.cn (Jie Wang)

(1) AHA oxidation degree and molecular weight results

AHA oxidation degree determinatio: Added 20 mL of 0.4 M hydroxylamine hydrochloride (NH_3OHCl) to 25 mL ultrapure water, and measured its pH as pH_0. A total of 60 mg of AHA was dissolved in 25 mL ultrapure water and adjusted the pH value of the solution to 7.0 with NaOH (0.01 M). Added 20 mL of 0.4 M NH_3OHCl to the solution, and stirred the reaction mixture at room temperature for more than 3 hours. Finally, titrated the mixed AHA solution with 0.01 M NaOH until it reached pH_0, and recorded the amount of NaOH solution. Repeated the titration three times for each sample. The content of the aldehyde group in AHA was calculated by the amount of NaOH, and the degree of hydroxyl substitution and oxidation in AHA was obtained.

(2) AHA molecular weight determination: 0.02 g AHA was dissolved in water. The molecular weight (MW) was measured on gel permeation chromatography (Agilent pl-gpc220, USA) with water as the mobile phase. The test results were as follows (Table S2). After the oxidation of sodium periodate, the molecular weight of AHA was significantly reduced.

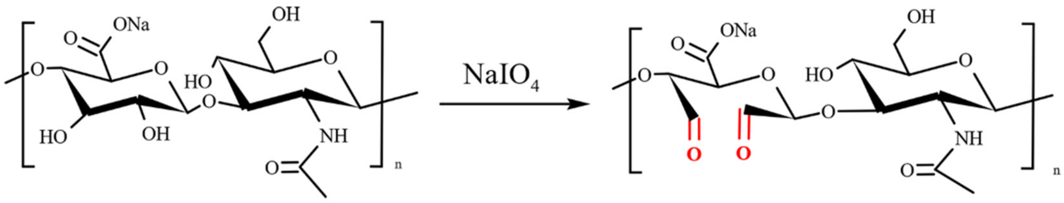

Figure S1. Synthesis route of AHA

Table S1. Reaction conditions for oxidation of native hyaluronic acid with the corresponding degrees of oxidation.

No.	Amount of $NaIO_4$ Used(eq)	Reaction Time(h)	n(CHO)/mmol	DO_{exp}(%) Titration
1	0.6	10	0.0035	0.55
2	0.6	24	0.0145	4.58
3	1.0	10	0.0253	7.97
4	1.0	24	0.0523	16.50
5	1.2	24	0.0779	24.59
6	1.2	72	0.1214	38.32

Table S2. GPC results of HA and AHA

Sample	Mw	PDI
HA	1724580	1.82
AHA-1	97521	1.70
AHA-2	63370	1.70
AHA-3	53482	1.62
AHA-4	27233	1.51
AHA-5	28870	1.56
AHA-6	18961	1.43

(3) Analysis of Dopa Substitution Degree in DAHA

Table S3. Synthesis of DAHA with different degrees of substitution(DS)

No.	DO_{AHA}	Feeding molar ratio DA: AHA	Reaction Time(h)	DS(%) by UV-vis
1	8	1.0	5	17.9
2	8	1.0	10	19.2
3	17	0.7	5	55.6
4	17	0.7	10	81.5
5	17	1.0	5	82.4
6	17	1.0	10	88.5
7	17	1.6	5	62.7
8	17	1.6	10	77.1

It could be seen from Table S3 that the degree of substitution of DA increased significantly when the oxidation degree of AHA (DO_{AHA}) or reaction time increased. And it could be seen that compared with oxidation degree and raw material ratio, reaction time has the least influence on it.

(4) DAHA infrared spectrum analysis

FTIR analysis of HA, AHA, and DAHA were carried out using an FTIR instrument (Nicolet 6700) at a scanning range from 4000 cm^{-1} to 500 cm^{-1} in Figure S3. Compared with AHA, the new small peak of DAHA at 1531 cm^{-1} was C=N stretching vibration, the increased peak at 1454 cm^{-1} was aromatic C-C stretching vibration and the new peak at 1285 cm^{-1} is C-N stretching vibration. FTIR results proved the existence of imine bond (-CH=N-) and aromatic ring, indicating that dopamine was successfully grafted onto HA molecular chain by using its own $-NH_2$ and the Schiff

base of the aldehyde group in AHA.

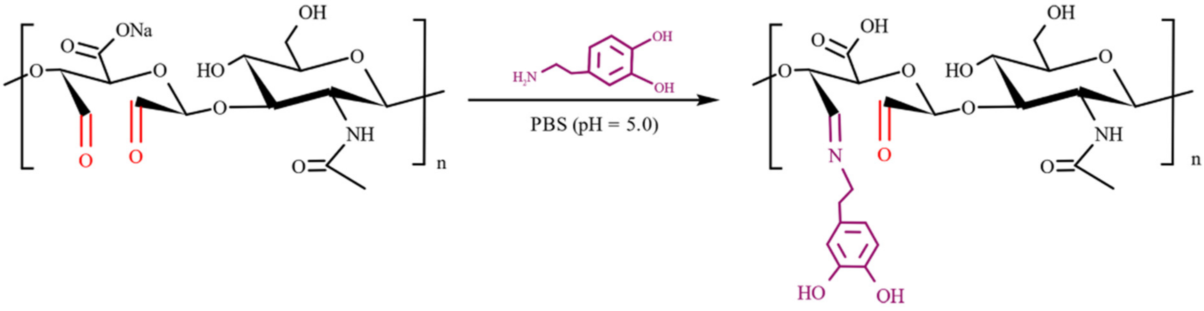

Figure S2. Synthesis route of DAHA

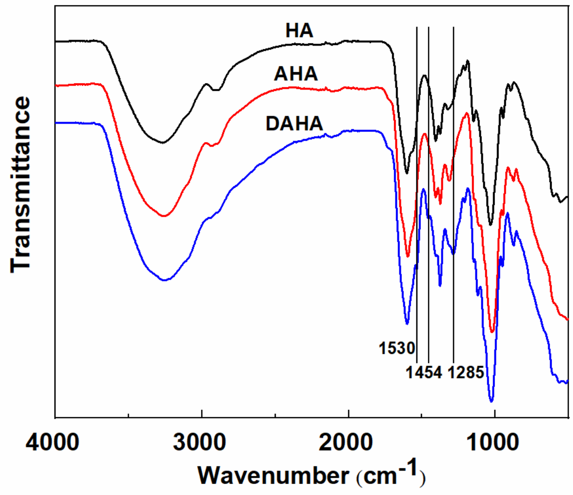

Figure S3. Infrared spectra of HA, AHA and DAHA

(5) DAHA ^{1}H NMR analysis

^{1}H NMR analysis of DA, HA, AHA, and DAHA were carried out using a ^{1}H NMR instrument (Ascend 600 M, BRUKER, USA) in Figure S4. AHA had three new chemical shifts b, c, and d between 4.6 ppm and 5.0 ppm, belonging to the proton peak of the aldehyde group in AHA. By comparing the nuclear magnetic spectrum of DAHA with the characteristic peak of AHA, it was found that a new characteristic peak of DAHA appeared at 6.7 ppm, which belonged to the proton

characteristic peak on the benzene ring. Both 2.8 ppm and 3.0 ppm on DAHA were the proton characteristic peaks of methylene on the DA chain. Therefore, ^{1}H NMR results showed that DA existed in AHA molecular chain, that was dopamine was successfully grafted onto AHA.

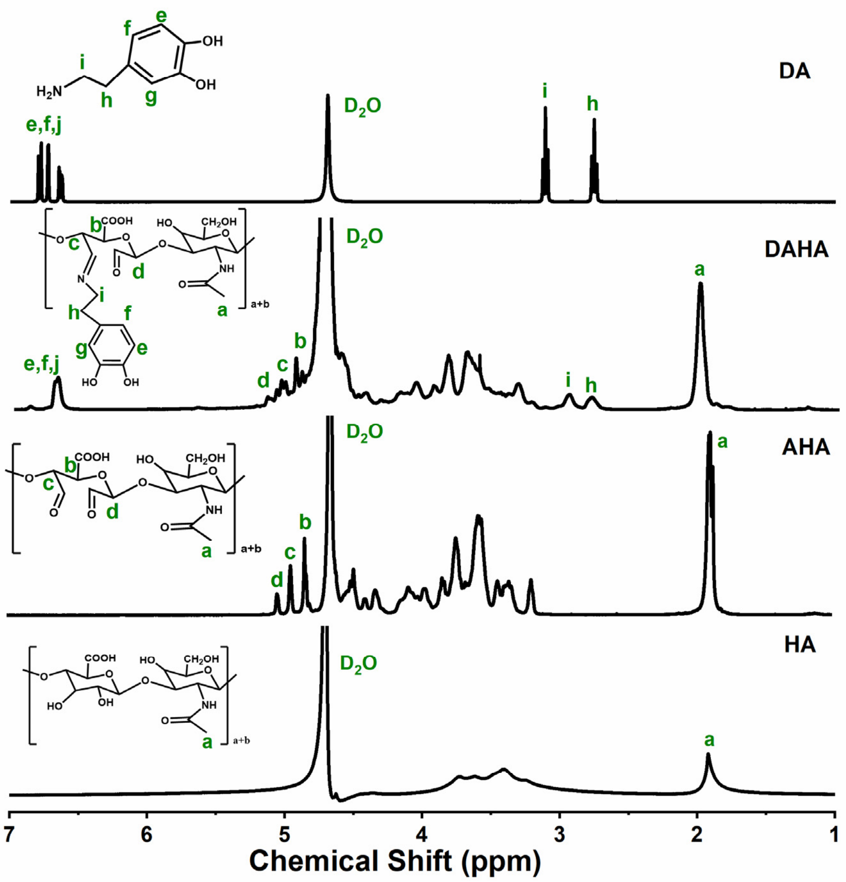

Figure S4. ^{1}H NMR spectra of DA, HA, AHA and DAHA

(6) DAHA ultraviolet spectrum analysis

The UV-vis spectra of DAHA were characterized by UV-1900i spectrophotometer (SHIMADZU) in Figure S5. DAHA had an obvious UV absorption peak at ~280 nm, which was the benzene ring structure absorption peak of the catechol group, which proved that the synthetic product DAHA contains a catechol structure.

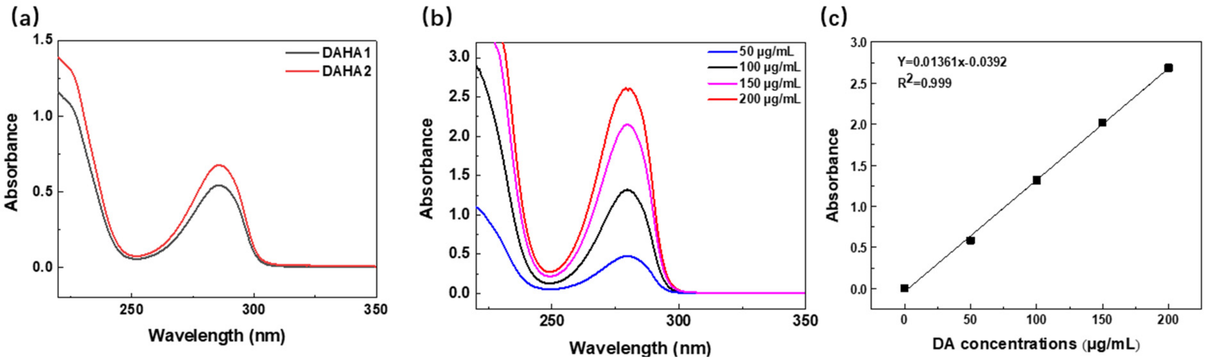

Figure S5. (**a**) UV-vis spectra of DAHA, (**b**) The absorbance of a series of dopamine hydrochloride solutions with different concentrations (ranging from 50 - 200 μg/mL) at 280 nm wavelength. (**c**) The standard curve generated by measuring the absorbance of a series of dopamine hydrochloride solution.
